# Differences in the content of five bioactive components in *Dendropanax dentiger* (Harms) Merr. from different production areas and their correlation with geographic distribution and climatic factors

**DOI:** 10.3389/fphar.2026.1702672

**Published:** 2026-01-21

**Authors:** Lan Lan, Jiale Mao, Weiqun Chen, Shuang Liu, Tao Li, Xiaohui Zhou, Tongtong Chen, Zhouxin Yuan, Houxing Lei, Zhen Hu, Zhengchun Zhang, Shaochang Wu, Xiaoqin Zhang

**Affiliations:** 1 School of Pharmaceutical Sciences, South-Central Minzu University, Wuhan, China; 2 Lishui TCM Hospital Affiliated to Zhejiang Chinese Medical University (Lishui Hospital of Traditional Chinese Medicine), Lishui, China; 3 Shaoxing Ruoxi Biotechnology Co., Ltd., Shaoxing, China; 4 Luo Yuan County Hospital of Traditional Chinese Medicine, Fuzhou, China

**Keywords:** bioactive constituents, climatic factors, *Dendropanax dentiger* (Harms) Merr., geographic distribution, multivariate statistical analysis

## Abstract

**Objective:**

Previous research on *Dendropanax dentiger* (Harms) Merr. has primarily focused on isolating and identifying its chemical constituents and evaluating pharmacological activities such as anti-inflammatory effects. This study aimed to characterize the geographical variation in the contents of five major bioactive components in roots across different regions and to examine their associations with geo-climatic factors.

**Methods:**

Chromatographic fingerprint analysis was performed on 23 batches of *D. dentiger* samples. Following chemometric analysis of the fingerprint data, five differential components, eleutheroside B, chlorogenic acid, saikolignanoside A, isochlorogenic acid A, and isochlorogenic acid C, were identified and subsequently quantified. The differences in the content of these components among samples from the five provinces were compared using one-way ANOVA. Furthermore, correlation and path analyses were performed to examine the relationships between the component contents and eight geographical-climatic variables.

**Results:**

The five bioactive components exhibited significant regional variation. Correlation analysis revealed the following relationships: The accumulation of eleutheroside B was correlated with precipitation in February, April, and December. Chlorogenic acid content showed correlations with precipitation in September, as well as with longitude and altitude within the studied range. Saikolignanoside A was correlated with precipitation in April, September, and December, solar radiation in April, and longitude. The content of isochlorogenic acid A correlated with precipitation in September, October, and December. Lastly, the accumulation of isochlorogenic acid C was associated with precipitation in February, April, September, October, and December.

**Conclusion:**

The content of five bioactive constituents in *D. dentiger* exhibited geographic variation across production areas, which was closely associated with precipitation in specific months, solar radiation, and geographical coordinates (longitude and altitude). These findings provide a reference for exploring the ecological causes of the *D. dentiger*’s chemical diversity and thus also for future studies.

## 1 Introduction

*Dendropanax* *dentiger* (Harms) Merr., commonly known as Shu-shen in She ethnic medicine, is a shrub or small tree belonging to the genus *Dendropanax* of the family Araliaceae. It has a long history of medicinal use, with documented efficacy in dispelling wind-dampness, promoting blood circulation, and reducing swelling. The herb is recorded in several authoritative Chinese materia medica references, including *Chinese Materia Medica*, *Dictionary of Chinese Materia Medica*, *National Compilation of Chinese Herbal Medicine*, and *Chinese She Ethnic Medicine* (Yang et al., 2021a; Balakrishnan et al., 2020). Traditionally, it has been prescribed for conditions such as rheumatism, arthralgia, soreness and weakness of the waist and legs, and general fatigue, and it is highly valued medicinally, earning the title “Ginseng of the She People.” With the continuous advancement of phytochemical and pharmacological studies, the therapeutic activities of *D. dentiger* have increasingly been recognized, particularly in anti-inflammatory, antioxidant, and immunoregulatory aspects (Balakrishnan et al., 2020; Wang Q. et al., 2025; Chien et al., 2014). However, research on its quality standards and the material basis of its pharmacological effects remains limited. Moreover, as *D. dentiger* is predominantly harvested from wild populations, considerable variability in raw material quality has hindered the development and clinical application of its medicinal resources. Notably, Wang Q. et al. (2025) employed affinity ultrafiltration coupled with liquid chromatography–mass spectrometry (AUF-LC-MS) to screen and identify ten phenylpropanoid compounds from *D. dentiger* roots, including chlorogenic acid, isochlorogenic acid A, eleutheroside E1, and isochlorogenic acid C. Molecular docking, molecular dynamics simulations, and enzyme inhibition assays further demonstrated that these compounds exert significant inhibitory activity against cyclooxygenase-2 (COX-2), thereby confirming their strong anti-inflammatory potential. Yang et al. (2021c) isolated a series of compounds from *Dendropanax dentiger*, among which the novel phenylpropanoids DRG and DAG were found to exert anti-inflammatory effects in TNF-α-induced MH7A cells by blocking the NF-κB, AKT, and JNK signaling pathways. Yang et al. (2020a) employed a bioassay-guided isolation method to investigate the roots of *Dendropanax dentiger*, leading to the discovery of phenylpropanoid derivatives with dual functions as COX-2 inhibitors and antioxidants.

*D*. *dentiger* is widely distributed across multiple provinces in China, including Zhejiang, Fujian, Jiangxi, Guangxi, and Hunan. Owing to differences in growth environments, the contents of its bioactive components may vary significantly among production areas. For instance, Su et al. (2023) reported that soil conditions play a critical role in the accumulation of citropten in citrus, while Yu et al. (2025) demonstrated that the levels of phenolic acids in *Salvia miltiorrhiza* differ significantly across production regions. However, current research on *D. dentiger* has mainly focused on chemical characterization and pharmacological activity (Yang et al., 2020b; Lai and Lee, 2013), whereas systematic investigations on the variation of bioactive constituents among different production areas remain scarce. Moreover, the correlations between these constituent levels and geographic or climatic factors have not yet been elucidated. Elucidating the regional variation in the contents of bioactive components in *D. dentiger* and their relationships with geographical and climatic parameters holds significant importance both for advancing scientific understanding and for guiding practical applications of this medicinal resource. Therefore, the present study compares the levels of five bioactive components—eleutheroside B, chlorogenic acid, saikolignanoside A, isochlorogenic acid A, and isochlorogenic acid C—in *D. dentiger* collected from Zhejiang, Fujian, Jiangxi, Guangxi, and Hunan provinces. Furthermore, based on the latitude, longitude, altitude, and climatic data of the sampling sites, correlation analysis and path analysis were applied to evaluate the influence of geographical and climatic factors on the accumulation of these compounds.

## 2 Materials

### 2.1 Instruments

PWN125DZH electronic balance (Ohaus International Trading Co., Ltd., Shanghai, China); XM-400UVF intelligent silent ultrasonic cleaner (Xiaomei ChaoSheng Instrument Co., Ltd., Kunshan, China); HWS-26 dual-row six-hole thermostatic water bath (Shanghai Yiheng Scientific Instrument Co., Ltd., Shanghai, China); Agilent 1260 high-performance liquid chromatography system (Agilent Technologies, United States); Lab-Q Direct ultrapure water purification system (Think-lab, Germany).

### 2.2 Reagents and reference standards

Protocatechuic acid (Batch No. 110809-201906, purity 97.7%) and chlorogenic acid (Batch No. 110753-202018, purity 96.1%) were purchased from the National Institutes for Food and Drug Control (Beijing, China). Eleutheroside B (Batch No. MUST-24061302), isochlorogenic acid A (Batch No. MUST-24102801), isochlorogenic acid C (Batch No. MUST-24032023), and sinapoyl glucoside (Batch No. MUST-25020704), each with purity ≥98%, were obtained from Chengdu Must Bio-Technology Co., Ltd. (Chengdu, China). Saikolignanoside A (Batch No. 103203, purity ≥95%) was supplied by Jiangsu Yongjian Pharmaceutical Technology Co., Ltd. (Jiangsu, China). Acetonitrile and formic acid (HPLC grade), methanol (analytical grade), and ultrapure water were used throughout the experiments.

**FIGURE 1:**
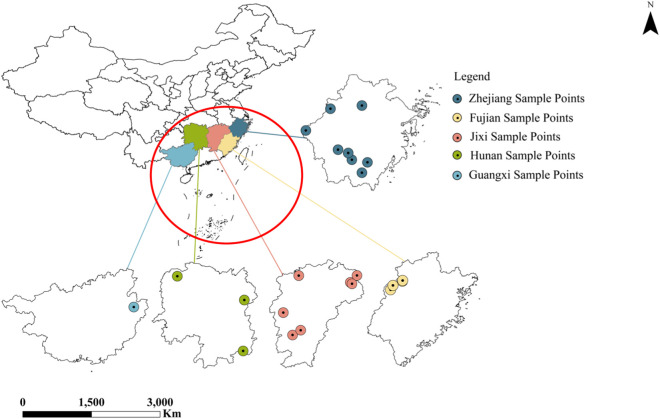
Map of the collection sample sites of *Dendropanax dentiger* *.* This map (Review Drawing No. GS (2020) 4619) is based on the standard map downloaded from the Standard Map Service website of the National Bureau of Surveying and Mapping Geographic Information (http://bzdt.ch.mnr.gov.cn/).

A total of 23 batches of *D*. *dentiger* crude drug samples from different production areas were collected (Figure 1; Table 1). All samples were collected from different *D. dentiger* individuals in October, during the same phenological stage. All specimens were authenticated by Chief Pharmacist Na Lin (Department of Traditional Chinese Medicine, Lishui Municipal Hospital of TCM, Zhejiang, China) as dried roots of *D. dentiger* (family Araliaceae). All samples were dried at 50 °C, pulverized, and sieved through a No. 4 mesh prior to use.

**TABLE 1:** Sample information of *D. dentiger*.

Sample no.	Collection site	Longitude/(°)	Latitude/(°)	Altitude/(m)	Sample size
S1	Anhou, Heqiao Town, Lin’an District, Hangzhou City, Zhejiang province	120.09	30.11	350.00	3
S2	Qingliangfeng Town, Lin’an District, Hangzhou City, Zhejiang province	119.00	30.00	420.00	3
S3	Wencheng County, Wenzhou City, Zhejiang province	120.09	27.79	760.00	3
S4	Yindai, Suichang County, Lishui City, Zhejiang province	119.26	28.59	500.00	3
S5	Dashanfeng Park, Liandu District, Lishui City, Zhejiang province	119.74	28.24	426.14	3
S6	Shangyuan Village, Songyang County, Lishui City, Zhejiang province	119.61	28.48	650.40	3
S7	Qingtian County, Lishui City, Zhejiang province	120.28	28.15	849.70	3
S8	Gutianshan, Kaihua County, Quzhou City, Zhejiang province	118.15	29.25	844.00	3
S9	Yufang, Zhukou Town, Taining County, Sanming City, Fujian province	117.30	27.10	350.00	3
S10	Suixi Town, Jianning County, Sanming City, Fujian province	116.81	26.75	330.00	3
S11	Huangfang Township, Jianning County, Sanming City, Fujian province	116.91	26.93	550.00	3
S12	Tongjiadi, Shaowu County, Nanping City, Fujian province	117.31	27.13	400.00	3
S13	Wuning County, Jiujiang City, Jiangxi province	115.10	29.26	500.00	3
S14	Wan’an County, Ji’an City, Jiangxi province	114.78	26.19	403.90	3
S15	Yian Highway, Anfu County, Ji’an City, Jiangxi province	114.30	27.35	308.00	3
S16	Chalong’an, Qingyuan District, Ji’an City, Jiangxi province	115.20	26.44	267.90	3
S17	Huangnishan, Dexing City, Jiangxi province	117.77	28.91	1076.00	3
S18	Yangjiaping, Dexing City, Jiangxi province	117.86	28.84	468.50	3
S19	Mushutan, Shangrao City, Jiangxi province	118.12	29.27	1141.80	3
S20	Poshan Peak, Zhaoping County, Hezhou City, Guangxi province	111.30	24.38	1289.90	3
S21	Zengjiawu, Liuyang City, Hunan province	113.57	28.17	191.90	3
S22	Guidong County, Chenzhou City, Hunan province	113.51	25.53	746.80	3
S23	Bamaoxi Township, Sangzhi County, Zhangjiajie City, Hunan province	110.10	29.40	375.80	3

### 2.3 Methods

#### 2.3.1 Fingerprint analysis of *D*. *dentiger*

##### 2.3.1.1 Preparation of sample solution

A total of 1.0 g of powdered *D. dentiger* root was accurately weighed and placed into a stoppered conical flask. Subsequently, 20 mL of 70% methanol was added, and the mixture was weighed and subjected to ultrasonic extraction for 30 min. After cooling, the weight was adjusted to the original mass with 70% methanol, mixed thoroughly, and filtered through a 0.22 μm microporous membrane to obtain the test solution.

##### 2.3.1.2 Preparation of reference solution

Appropriate amounts of protocatechuic acid, eleutheroside B, chlorogenic acid, sinapoyl glucoside, saikolignanoside A, isochlorogenic acid A, and isochlorogenic acid C reference substances were accurately weighed and dissolved in methanol. A mixed standard solution was prepared with final concentrations of 0.0770, 0.1395, 0.2060, 0.0269, 0.0950, 0.3650, and 0.1050 mg/mL, respectively, and then filtered through a 0.22 μm microporous membrane for subsequent use.

##### 2.3.1.3 Chromatographic conditions

Chromatographic separation was carried out on a Waters XBridge C18 column (250 mm × 4.6 mm, 5 μm). The detection wavelength was set at 260 nm, the flow rate at 1.0 mL/min, and the column temperature at 35 °C, with an injection volume of 10 μL. The mobile phase consisted of acetonitrile (A) and 0.5% aqueous formic acid (B), with the following gradient program: 0–13 min, 11%–11% A; 13–32 min, 11%–35% A; 32–42 min, 35%–42% A; and 42–45 min, 42%–11% A.

##### 2.3.1.4 Selection of reference peak

Peak 3 (chlorogenic acid) was chosen as the reference peak (S) because it showed excellent separation, a stable peak shape, a smooth baseline, and a relatively large peak area. It was therefore used to calculate the relative retention times and relative peak areas of all common peaks.

##### 2.3.1.5 Precision test

The powdered sample S5 was prepared according to the method described in Section 2.3.1.1, and the test solution was analyzed consecutively six times under the chromatographic conditions specified in Section 2.3.1.3. The relative standard deviations (RSDs) of relative retention times were ≤0.04%, and those of relative peak areas were ≤2.14%, indicating good instrumental precision.

##### 2.3.1.6 Repeatability test

Six aliquots of the powdered sample S5 were prepared in parallel according to Section 2.3.1.3, and each was analyzed under the chromatographic conditions described in Section 2.1.3. The RSDs of relative retention times were ≤0.02%, and those of relative peak areas were ≤1.96%, demonstrating satisfactory method repeatability.

##### 2.3.1.7 Stability test

A test solution of sample S5 was prepared as described in Section 2.3.1.1, and chromatograms were recorded at 0, 2, 4, 8, 12, 16, and 24 h under the conditions specified in Section 2.3.1.3. The RSDs of relative retention times were ≤0.05%, and those of relative peak areas were ≤2.01%, indicating that the solution remained stable within 24 h.

##### 2.3.1.8 Establishment of fingerprints and similarity evaluation

The HPLC chromatographic data of 23 batches of *D. dentiger* samples were imported into the *Similarity Evaluation System for Chromatographic Fingerprints of Traditional Chinese Medicines* (2012 edition). The chromatogram of sample S5 was used as the reference, with a time window of 0.5 min. After multipoint correction using the mean method, peak matching was performed with marker peaks, and superimposed chromatograms, as well as reference fingerprints, were generated for all samples.

### 2.4 Chemometric analysis of 23 batches of *D. dentiger*

#### 2.4.1 Cluster analysis

The peak areas of eight common peaks from 23 batches of *D. dentiger* samples were used as variables and imported into SPSS 25.0 software. Hierarchical Cluster analysis was performed using the between-groups linkage method and squared Euclidean distance, and a dendrogram was generated.

#### 2.4.2 Principal component analysis (PCA)

The peak areas of eight common peaks from the fingerprint chromatograms of 23 batches of *D. dentiger* were imported into SIMCA 14.1 and SPSS 25.0 software for PCA analysis, and PCA score plots were constructed. Principal components were extracted according to the criterion of eigenvalues greater than 1. The eigenvalues, variance contribution rates, and cumulative variance contribution rates of each principal component were calculated to evaluate the representativeness of the extracted information. A factor loading matrix was generated by the software, in which the numerical values represented the correlation coefficients between each principal component and the eight original common peak variables. Based on this matrix, the original peak information primarily reflected by each principal component was identified.

#### 2.4.3 Orthogonal partial least squares discriminant analysis (OPLS-DA)

The peak areas of eight common peaks from 23 batches of samples were imported into SIMCA 14.1 software, and an OPLS-DA model was established for analysis. As a supervised discriminant statistical method, OPLS-DA effectively reflects intergroup differences (Boccard and Rutledge, 2013). To pinpoint the compounds responsible for the observed interregional differences, variable importance in projection (VIP) values were calculated. The VIP metric summarizes the contribution of each variable (compound peak) to the OPLS-DA model. Compounds with a VIP value greater than 1.0 were considered significantly influential for the discrimination. The stability and reliability of the model were evaluated using model parameters R^2^X, R^2^Y, and Q^2^, where R^2^X represents the explanatory ability for X variables, R^2^Y represents the explanatory ability for Y variables, and Q^2^ reflects the predictive ability of the model. Sample classification was analyzed based on the model score plot. Differential compounds were screened with VIP >1.0 set as the threshold, where higher VIP values indicate greater contributions to intergroup differentiation (Wang et al., 2024b).

### 2.5 Determination of five components in *D*. *dentiger*

#### 2.5.1 Preparation of sample solution

The sample solution was prepared as described in Section 2.3.1.1.

#### 2.5.2 Preparation of reference solution

Appropriate amounts of eleutheroside B, chlorogenic acid, saikolignanoside A, isochlorogenic acid A, and isochlorogenic acid C reference substances were accurately weighed and dissolved in methanol. A mixed reference solution was prepared with final concentrations of 0.1395, 0.2060, 0.0950, 0.3650, and 0.1050 mg/mL, respectively. The solution was filtered through a 0.22 μm microporous membrane for subsequent use.

#### 2.5.3 Chromatographic conditions

Chromatographic conditions were the same as those described in Section 2.3.1.3.

#### 2.5.4 Linearity study

The mixed reference solution prepared in Section 2.5.2 was serially diluted with methanol to obtain solutions of different concentrations. Each solution was analyzed under the chromatographic conditions described in Section 3.1.3. Calibration curves were constructed by plotting peak area (Y) against concentration (X). Linear regression analysis was performed to obtain equation (Y = aX + b) and the coefficient of determination (*R* ^2^) for each compound, ensuring a good linear fit within the tested range.

#### 2.5.5 Precision study

A test solution of sample S5 was prepared according to Section 2.3.1.1 and injected consecutively six times under the chromatographic conditions described in Section 2.3.1.3. The peak areas of eleutheroside B, chlorogenic acid, saikolignanoside A, isochlorogenic acid A, and isochlorogenic acid C were recorded. The RSD values were 1.42%, 0.38%, 0.26%, 0.62%, and 0.09%, respectively, indicating good instrumental precision.

#### 2.5.6 Repeatability test

Six aliquots of the same batch of sample S5 were prepared in parallel according to Section 2.3.1.1, and each was analyzed under the chromatographic conditions specified in Section 2.3.1.3. The RSD values of relative peak areas for eleutheroside B, chlorogenic acid, saikolignanoside A, isochlorogenic acid A, and isochlorogenic acid C were 2.38%, 2.55%, 2.11%, 2.19%, and 2.77%, respectively, demonstrating good method repeatability.

#### 2.5.7 Stability test

A test solution of sample S5 was prepared according to Section 2.3.1.1 and analyzed at 0, 2, 4, 8, 12, 16, and 24 h under the chromatographic conditions described in Section 2.3.1.3. The RSD values of relative peak areas for eleutheroside B, chlorogenic acid, saikolignanoside A, isochlorogenic acid A, and isochlorogenic acid C were 1.23%, 0.52%, 0.26%, 0.12%, and 0.20%, respectively, indicating that the test solution was stable within 24 h.

#### 2.5.8 Recovery test

Six aliquots of the same batch of previously analyzed samples were spiked with appropriate amounts of reference substances. The spiked samples were prepared according to Section 2.3.1.1 and analyzed under the chromatographic conditions described in Section 2.3.1.3. The average recoveries of eleutheroside B, chlorogenic acid, saikolignanoside A, isochlorogenic acid A, and isochlorogenic acid C were 101.46%, 101.90%, 98.44%, 102.70%, and 102.08%, with RSD values of 2.53%, 2.53%, 2.61%, 2.22%, and 2.27%, respectively, which met the methodological requirements.

#### 2.5.9 Sample determination

Powdered samples were prepared into test solutions according to Section 2.3.1.1, with three replicates for each batch. The solutions were analyzed under the chromatographic conditions specified in Section 2.3.1.3, and the contents of eleutheroside B, chlorogenic acid, saikolignanoside A, isochlorogenic acid A, and isochlorogenic acid C in each batch of *D. dentiger* samples were calculated using the external standard method.

### 2.6 Data collection and statistical methods

Meteorological data were obtained from the global climate database (https://www.worldclim.org), and annual mean temperature, annual precipitation, and monthly mean temperature, precipitation, and solar radiation from April to October for each production area were extracted using ArcGIS 10.2 software. Latitude and longitude, as well as altitude, were measured through field surveys. In total, 41 geographic and climatic variables were collected. The contents of five components—eleutheroside B, chlorogenic acid, saikolignanoside A, isochlorogenic acid A, and isochlorogenic acid C—were determined by HPLC. The resulting data were organized and analyzed using Microsoft Excel 2013, and subsequently imported into SPSS 27.0 for one-way analysis of variance (ANOVA). To identify which specific production areas differed significantly from each other following a significant ANOVA result, multiple comparisons were conducted using the Student-Newman-Keuls (S-N-K) test. In addition, to dissect the direct and indirect effects of the geographical-climatic factors on component contents, correlation analysis was followed by path analysis using SPSS 27.0. In addition, to dissect the direct and indirect effects of the geographical-climatic factors on component contents, correlation analysis was followed by path analysis using SPSS 27.0.

## 3 Results and analysis

### 3.1 Establishment of fingerprints and similarity evaluation

**FIGURE 2:**
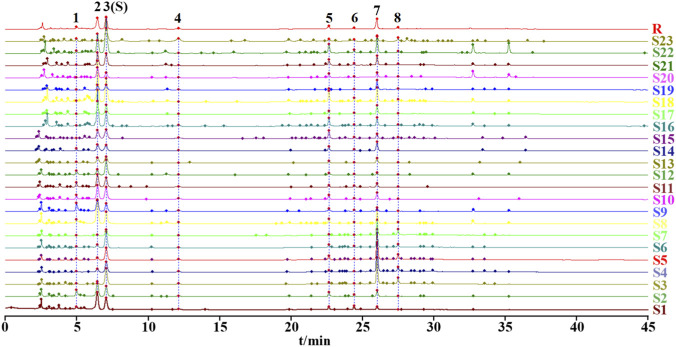
HPLC fingerprints of 23 batches of *D. dentiger* and control fingerprints (R).

**FIGURE 3:**
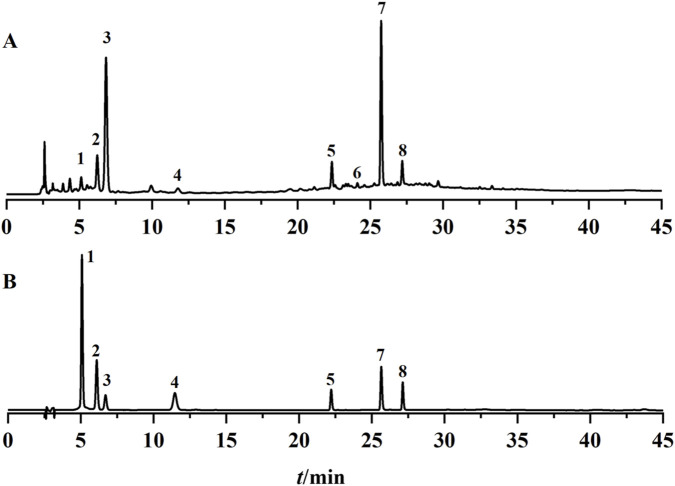
HPLC chromatograms of *Dendropanax dentiger* sample **(A)** and mixed reference standards **(B)**. 1 – protocatechuic acid; 2 – eleutheroside B; 3 – chlorogenic acid; 4 – sinapoyl glucoside; 5 – saikolignanoside A; 7 – isochlorogenic acid A; 8 – isochlorogenic acid C.

The HPLC chromatographic files of 23 batches of *D*. *dentiger* samples were imported into the *Similarity Evaluation System for Chromatographic Fingerprints of Traditional Chinese Medicines* (2012 edition). Overlaid HPLC chromatograms and a reference fingerprint (R) were generated (Figure 2), revealing eight common peaks. Seven of these peaks were unambiguously identified by matching both their retention times and full-wavelength UV absorption spectra with those of authentic reference standards: Peak 1 was protocatechuic acid, Peak 2 was eleutheroside B, Peak 3 was chlorogenic acid, Peak 4 was sinapoyl glucoside, Peak 5 was saikolignanoside A, Peak 7 was isochlorogenic acid A, and Peak 8 was isochlorogenic acid C (Figure 3). Using the generated reference chromatogram (R) as the standard, the similarities of 23 batches of *D. dentiger* samples were calculated with the *Similarity Evaluation System for Chromatographic Fingerprints of Traditional Chinese Medicines* (2012 edition). The similarities varied between 0.736 and 0.997 (Table 2). Specifically, batches S3, S4, S5, and S7 had similarities of 0.783, 0.758, 0.785, and 0.736, respectively, whereas the remaining batches all exceeded 0.86. These results indicate notable quality variations among the different batches of samples.

**TABLE 2:** Results of fingerprint similarity evaluation for 23 batches of *D. dentiger* samples.

Sample no.	Similarity	Sample no.	Similarity
S1	0.926	S13	0.997
S2	0.954	S14	0.979
S3	0.783	S15	0.958
S4	0.758	S16	0.879
S5	0.785	S17	0.982
S6	0.868	S18	0.940
S7	0.736	S19	0.943
S8	0.958	S20	0.936
S9	0.917	S21	0.991
S10	0.976	S22	0.955
S11	0.945	S23	0.791
S12	0.881	​	​

### 3.2 Cluster analysis of 23 batches of *D. dentiger*

**FIGURE 4:**
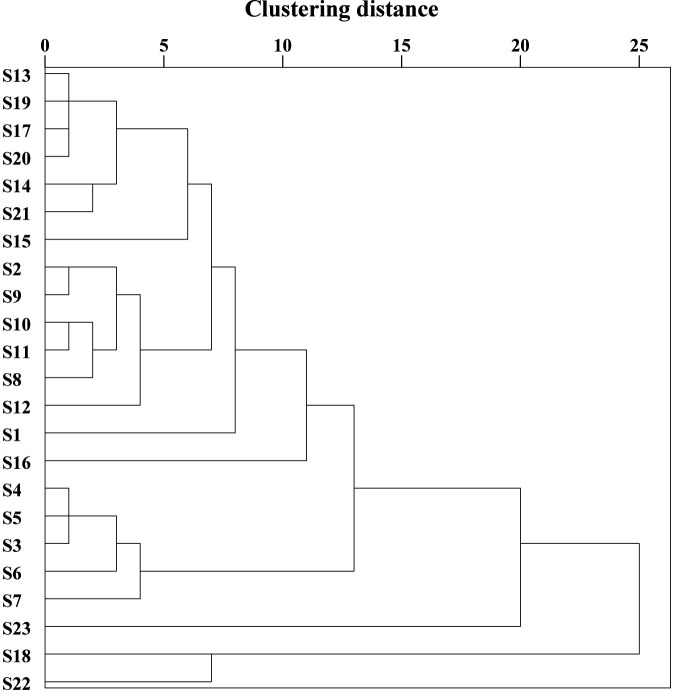
Cluster analysis diagram of 23 batches of *D. dentiger* samples.

The dendrogram of 23 batches of *D*. *dentiger* samples is shown in Figure 4. At a clustering distance of 15, the samples were divided into three groups: S18 and S22 clustered together as one group, S23 formed a separate group, and the remaining samples clustered into another group. When the distance was reduced to 10, the 23 batches were divided into five groups. Group 1 included S1 (Anhou, Zhejiang), S2 (Qingliangfeng, Zhejiang), S8–S15 (Gutianshan, Zhejiang; Yufang, Suixi, Huangfang and Tongjiadi in Fujian; Wuning,Wan’an and Yian in Jiangxi), S17 (Chalongan, Jiangxi) and S19–S21 (Mushutan, Jiangxi; Poshan Peak, Guangxi; Zengjiawu, Hunan), covering 14 production areas. Group 2 included S3–S7 (Wencheng, Yindai, Dashanfeng Park, Shangyuan and Qingtian in Zhejiang), covering five production areas; Group 3 consisted of S18 (Yangjiaping, Jiangxi) and S22 (Guidong, Hunan), originating from two different production areas; while S16 (Chalong’an, Jiangxi) and S23 (Bamaoxi, Hunan) each formed independent groups. The cluster analysis indicated that there were significant quality differences among *D. dentiger* samples from different production areas. While samples from the same region generally clustered together, some quality variation was still evident within the same origin, which may be attributed to the wild-growimg nature of *D. dentiger*.

### 3.3 PCA of 23 batches of *D. dentiger*

**FIGURE 5:**
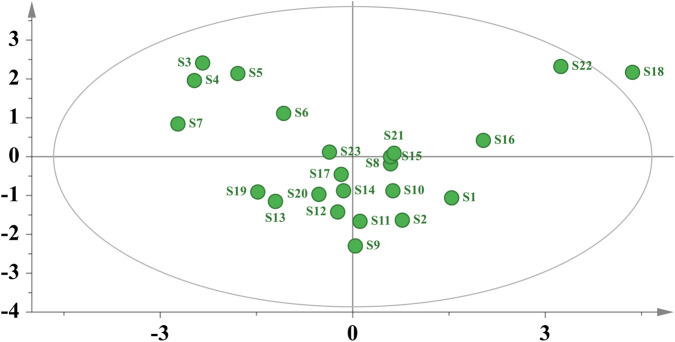
PCA score plot of 23 batches of *D. dentiger* samples.

PCA is a statistical method that reduces the dimensionality of data by transforming multiple intercorrelated variables into a smaller number of uncorrelated comprehensive indicators while minimizing information loss (Jin et al., 2016). The PCA score plot is shown in Figure 5, in which S1 (Anhou, Zhejiang), S2 (Qingliangfeng, Zhejiang), S8–S15 (Gutianshan, Zhejiang; Yufang, Suixi, Huangfang and Tongjiadi in Fujian; Wuning,Wan’an and Yian in Jiangxi), S17 (Chalongan, Jiangxi), and S19–S21 (Mushutan, Jiangxi; Poshan Peak, Guangxi; Zengjiawu, Hunan) were relatively clustered, while S3–S7 (Wencheng, Yindai, Dashanfeng Park, Shangyuan and Qingtian in Zhejiang) were also relatively clustered. This indicated that substantial quality differences existed among the 23 batches of *D*. *dentiger* samples, which were generally consistent with the CA results.

Using the peak areas of the common peaks from the HPLC fingerprints of the 23 batches of *D. dentiger* samples as variables, factor analysis was performed in SPSS 25.0 through dimensionality reduction. Three principal components with eigenvalues greater than 1 were extracted, and their identities were interpreted based on the factor loadings as being primarily associated with protocatechuic acid, eleutheroside B, and chlorogenic acid, respectively. The cumulative variance contribution rate was 78.074%. This suggested that these three principal components were able to represent most of the information contained in the eight common peaks of the 23 samples, with high representativeness. The first principal component mainly reflected information from Peaks 2 (eleutheroside B), 3 (chlorogenic acid), 5 (saikolignanoside A), and 6; the second principal component mainly reflected information from Peaks 3 (chlorogenic acid), 7 (isochlorogenic acid A), and 8 (isochlorogenic acid C); and the third principal component mainly reflected information from Peaks 1 (protocatechuic acid) and 4 (sinapoyl glucoside). Therefore, these three principal components could be used as substitutes for the eight common peaks of the fingerprint to evaluate *D. dentiger*. The eigenvalues and contribution rates are presented in Table 3. The factor loading matrix reflected the correlation coefficients between each principal component and the eight common peaks (original variables), and the results are shown in Table 4.

**TABLE 3:** Eigenvalue and variance contribution rate.

Principal component	Eigen value	Variance contribution (%)	Cumulative variance contribution (%)
1	2.991	37.386	37.386
2	2.054	25.681	63.067
3	1.201	15.007	78.074

**TABLE 4:** Factor load matrix.

Common peak no. (compound)	Principal component loadings		
	1	2	3
Peak 2 (Eleutheroside B)	0.857	0.043	−0.135
Peak 5 (Saikolignanoside A)	0.852	0.348	−0.064
Peak 6	0.829	0.062	0.196
Peak 3 (Chlorogenic acid)	0.646	0.582	0.115
Peak 1 (Protocatechuic acid)	0.128	−0.560	0.467
Peak 4 (Sinapaldehyde glucoside)	0.033	0.005	0.902
Peak 7 (Isochlorogenic acid A)	−0.344	0.853	−0.026
Peak 8 (Isochlorogenic acid C)	−0.536	0.740	0.308

### 3.4 OPLS-DA of 23 batches of *D. dentiger*

**FIGURE 6:**
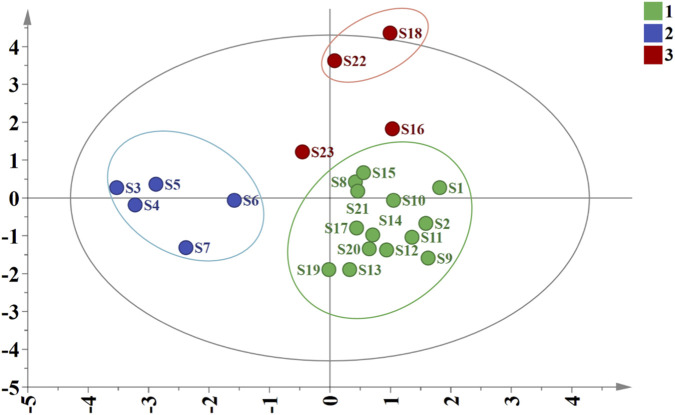
OPLS-DA score plot of 23 batches of *D. dentiger* samples.

**FIGURE 7:**
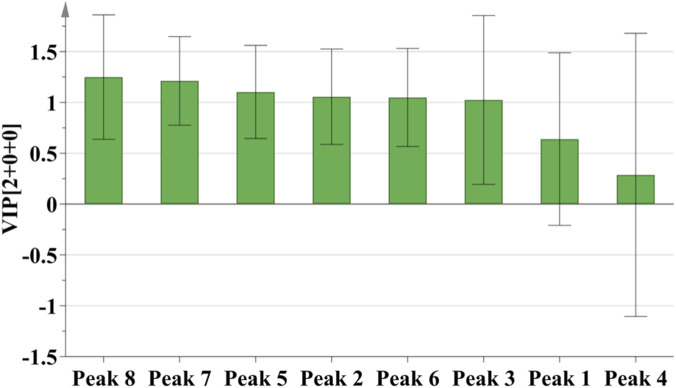
VIP values of 8 common peaks of 23 batches of *D. dentiger* samples.

The OPLS-DA results showed that the model parameters R^2^X and R^2^Y were 0.622 and 0.772, respectively, and the predictive ability parameter Q^2^ was 0.65 (Q2 > 0.5 is generally considered indicative of a good predictive model). These values indicated good model stability and reliability. As shown in the score plot of the model (Figure 6), the 23 batches of samples were mainly divided into three groups, with S16 and S23 each forming independent groups, which was largely consistent with the Cluster analysis results when the clustering distance was set at 10. VIP values were calculated to identify differential compounds. The VIP metric quantifies each variable’s contribution to the group discrimination in the model. Based on a threshold of VIP >1.0, six differential compounds were identified (Figure 7). These included Peak 8 (isochlorogenic acid C), Peak 7 (isochlorogenic acid A), Peak 5 (saikolignanoside A), Peak 2 (eleutheroside B), Peak 6, and Peak 3 (chlorogenic acid). From these, the five authentically identified compounds: isochlorogenic acid C, isochlorogenic acid A, saikolignanoside A, eleutheroside B, and chlorogenic acid, were selected for subsequent quantification.

### 3.5 Linearity assessment of the five components in *D*. *dentiger*

The calibration curves of the five components—eleutheroside B, chlorogenic acid, saikolignanoside A, isochlorogenic acid A, and isochlorogenic acid C—are shown in Table 5. The correlation coefficients (*R* ^2^) were all ≥0.999, indicating that good linear relationships were obtained for all components within their respective concentration ranges.

**TABLE 5:** Linear regression equations of five components in *Dendropanax dentiger*.

Compound	Regression equation	*R* ^2^	Linear range/μg
Eleutheroside B	y = 3518.8x - 455.71	0.9992	0.1367–0.6836
Chlorogenic acid	y = 1093.9x + 44.816	0.9928	0.1980–0.9898
Saikolignanoside A	y = 1785.3x + 11.17	0.9990	0.0950–0.5700
Isochlorogenic acid A	y = 999.23x + 22.928	0.9994	0.3586–2.1515
Isochlorogenic acid C	y = 1209.1x + 12.402	0.9990	0.1046–0.8367

### 3.6 Differential analysis of bioactive component contents in *D. dentiger* from different production areas

**FIGURE 8:**
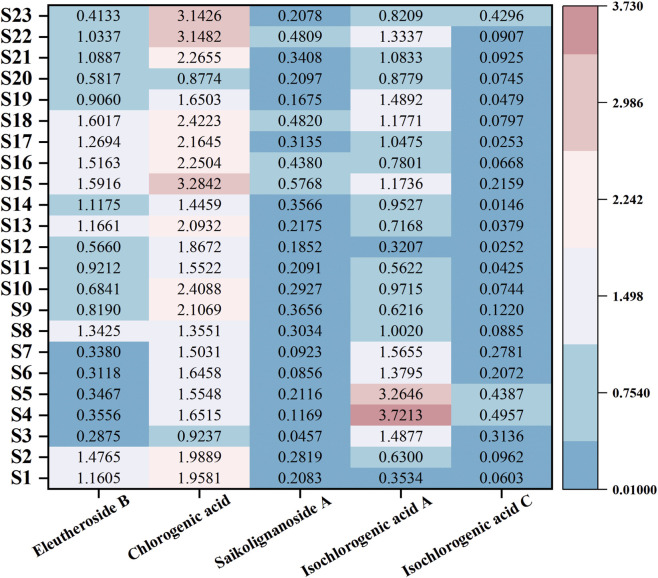
Heat map of the content of five active ingredients in 23 batches of *D. dentiger* samples.

A heat map was used as a statistical method to visualize the results, with a progressive color scale where deeper red indicated higher contents of bioactive components. As shown in Figure 8, the content of eleutheroside B in the 23 batches of *D. dentiger* ranged from 0.2875 to 1.6017 mg/g, with the highest level observed in S18. The content of chlorogenic acid ranged from 0.8774 to 0.3824 mg/g, with relatively higher levels in S15, S22, and S23, and the highest in S15. The content of saikolignanoside A ranged from 0.0457 to 0.5768 mg/g, with the highest observed in S15. The content of isochlorogenic acid A ranged from 0.3207 to 0.3713 mg/g, while that of isochlorogenic acid C ranged from 0.0252 to 0.4957 mg/g, with S4 and S5 exhibiting higher levels of both isochlorogenic acid A and isochlorogenic acid C. Overall, S15 (Anfu, Jiangxi Province) exhibited the highest combined contents of eleutheroside B, chlorogenic acid, and saikolignanoside A, while S4 (Suichang, Zhejiang Province) exhibited the highest combined contents of isochlorogenic acid A and isochlorogenic acid C.

Significant differences were observed in the contents of the five bioactive components among samples from different provinces (Table 6). The contents of isochlorogenic acid A and isochlorogenic acid C were highest in Zhejiang Province, with isochlorogenic acid A being significantly higher than those in Fujian and Jiangxi, and isochlorogenic acid C being significantly higher than those in Fujian, Jiangxi, and Guangxi. The contents of eleutheroside B and saikolignanoside A were highest in Jiangxi Province, significantly exceeding those in Zhejiang, Fujian, Guangxi, and Hunan. The content of chlorogenic acid was highest in Hunan Province, significantly higher than that in Zhejiang, Fujian, Jiangxi, and Guangxi. In summary, the total contents of eleutheroside B, chlorogenic acid, and saikolignanoside A were relatively higher in Jiangxi Province, whereas the total contents of isochlorogenic acid A and isochlorogenic acid C were higher in Zhejiang Province, which was consistent with the results shown in Figure 8.

**TABLE 6:** Analysis of variance of contents of six components in *D. dentiger* from different regions (x̄ ± s).

Production area	Content/(mg/g)				
	Eleutheroside B	Chlorogenic acid	Saikolignanoside A	Isochlorogenic acid A	Isochlorogenic acid C
Zhejiang	0.69 ± 0.52a	1.58 ± 0.67a	0.17 ± 0.1a	1.76 ± 1.42a	0.26 ± 0.2a
Fujian	0.75 ± 0.22b	1.98 ± 0.72b	0.26 ± 0.13	0.62 ± 0.29a	0.07 ± 0.06ab
Jiangxi	1.31 ± 0.54abc	2.19 ± 1.13ac	0.36 ± 0.18a	1.05 ± 0.39a	0.07 ± 0.08ac
Guangxi	0.58 ± 0.16c	0.88 ± 0.3cd	0.21 ± 0.1	0.88 ± 0.38	0.07 ± 0.02a
Hunan	0.85 ± 0.51c	2.85 ± 1.38abd	0.34 ± 0.14a	1.08 ± 0.4	0.2 ± 0.17bc
*F*	5.73	4.38	6.50	3.82	6.57
*P*	<0.001	0.003	<0.001	0.008	<0.001

### 3.7 Correlation analysis between bioactive component contents and geographic–climatic factors

Correlation analysis was performed between the content of five chemical components and 41 geographical and climatic factors (including mean annual temperature, annual precipitation, monthly mean temperature, monthly precipitation, and solar radiation data from April to October, as well as longitude, latitude, and altitude of each production area) obtained in Section 2.6. The correlation coefficients were ranked according to their absolute values, and the top three geographic–climatic factors associated with each component were screened. Collinearity diagnostics were then performed, and all eigenvalues for collinearity tests were less than 5. A total of eight key factors were finally selected: longitude (X1), altitude (X2), February precipitation (X3), April precipitation (X4), September precipitation (X5), October precipitation (X6), December precipitation (X7), and April solar radiation (X8).

**FIGURE 9:**
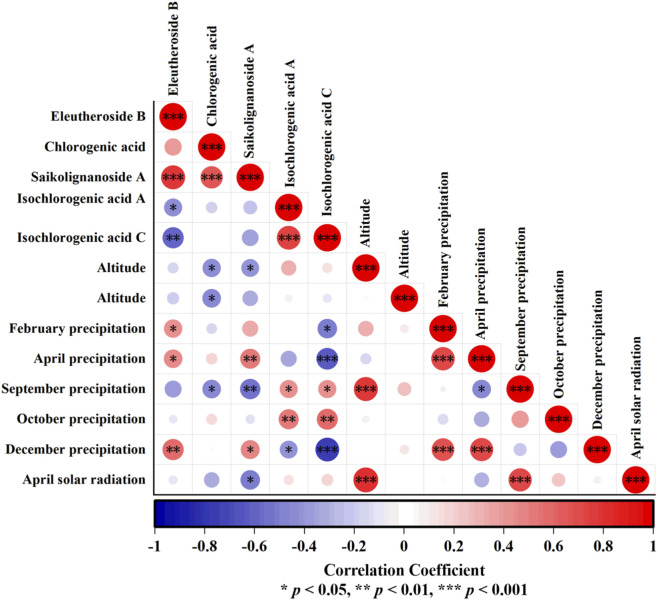
Heat map of correlation between 6 component contents and key climate factors.

Correlation analyses between the five components and these eight factors (Figure 9) showed that isochlorogenic acid A and isochlorogenic acid C were significantly positively correlated with precipitation in September and October, and significantly negatively correlated with precipitation in December. Isochlorogenic acid C was also significantly negatively correlated with precipitation in February and April. Chlorogenic acid and saikolignanoside A were significantly negatively correlated with longitude and precipitation in September. Eleutheroside B and saikolignanoside A were significantly positively correlated with precipitation in April and December. In addition, chlorogenic acid was negatively correlated with altitude, saikolignanoside A was negatively correlated with solar radiation in April, and eleutheroside B was positively correlated with precipitation in February.

### 3.8 Path analysis between bioactive component contents and geographic–climatic factors

Complex interactions existed among the geographic–climatic factors, and mutual influences were also observed between different climatic variables. However, such effects could not be fully explained by correlation coefficients alone. Path analysis was therefore applied to further partition the correlation coefficients and to clarify causal relationships among the data. This method was used to evaluate both the direct and indirect effects of geographic–climatic factors on the accumulation of bioactive components (Zhang et al., 2019). The absolute values of the path coefficients were used to measure the relative importance of the corresponding geographic–climatic factors during component accumulation, with direct and indirect path coefficients reflecting direct and indirect effects, respectively.

The path analysis of the five components with eight geographic–climatic factors was conducted. The results (Table 7) showed that longitude exerted a positive effect on the accumulation of isochlorogenic acid A and isochlorogenic acid C, while it exerted a negative effect on the accumulation of eleutheroside B, chlorogenic acid, and saikolignanoside A. The direct path coefficients were 0.5224, 0.0432, −0.2930, 0.9970, and −0.0416, respectively, whereas the indirect path coefficients were −0.1997, 0.0977, 0.1288, −1.4498, and −0.3894, respectively. Notably, longitude influenced the accumulation of chlorogenic acid primarily through indirect effects, with the absolute value of the indirect path coefficient exceeding that of the direct path coefficient, indicating a pronounced overall negative influence. Altitude exhibited a weak positive effect on the accumulation of isochlorogenic acid A, while exerting negative effects on the accumulation of eleutheroside B, chlorogenic acid, saikolignanoside A, and isochlorogenic acid C. The direct path coefficients were 0.0293, −0.2104, −0.3038, −0.4130, and −0.1551, respectively, and the indirect path coefficients were 0.0596, 0.0100, −0.1638, 0.0807, and 0.0400, respectively, and the corresponding indirect path coefficients were 0.0596, 0.0100, −0.1638, 0.0807, and 0.0400. Altitude primarily influenced the accumulation of eleutheroside B, saikolignanoside A, and isochlorogenic acid C through direct effects, as the absolute values of the direct path coefficients were greater than those of the indirect path coefficients.

**TABLE 7:** Path analysis results of the content of medicinal ingredients and geographical climate factors.

Component	Geographic–climatic factor	Direct path coefficient	Indirect path coefficient								
			X1	X2	X3	X4	X5	X6	X7	X8	Total
Eleutheroside B	X1	−0.2930	​	−0.0119	0.1356	0.0573	−0.2934	0.0164	0.0055	0.2195	0.1288
	X2	−0.2104	−0.0166	​	0.0444	−0.0032	−0.0988	0.0075	0.0777	−0.0011	0.0100
	X3	0.4079	−0.0974	−0.0229	​	−0.2486	0.0172	−0.0316	0.4177	0.0148	0.0493
	X4	−0.3372	0.0498	−0.0020	0.3007	​	0.1742	−0.0651	0.4332	−0.0802	0.8107
	X5	−0.3737	−0.2301	−0.0556	−0.0188	0.1572	​	0.0807	−0.1387	0.1911	−0.0142
	X6	0.2013	−0.0238	−0.0078	−0.0640	0.1091	−0.1498	​	−0.2337	0.0657	−0.3044
	X7	0.6005	−0.0027	−0.0272	0.2837	−0.2433	0.0863	−0.0784	​	−0.0199	−0.0014
	X8	0.2650	−0.2426	0.0009	0.0228	0.1020	−0.2695	0.0499	−0.0450	​	−0.3814
Chlorogenic acid	X1	0.9970	​	−0.0172	−0.3602	−0.0794	−0.6210	0.0609	0.0051	−0.4380	−1.4498
	X2	−0.3038	0.0564	​	−0.1181	0.0044	−0.2091	0.0279	0.0724	0.0023	−0.1638
	X3	−1.0838	0.3313	−0.0331	​	0.3444	0.0364	−0.1174	0.3889	−0.0296	0.9210
	X4	0.4673	−0.1694	−0.0028	−0.7989	​	0.3688	−0.2422	0.4034	0.1600	−0.2812
	X5	−0.7909	0.7829	−0.0803	0.0499	−0.2179	​	0.3002	−0.1292	−0.3814	0.3243
	X6	0.7489	0.0811	−0.0113	0.1700	−0.1511	−0.3170	​	−0.2177	−0.1311	−0.5772
	X7	0.5591	0.0091	−0.0393	−0.7539	0.3371	0.1827	−0.2915	​	0.0396	−0.5161
	X8	−0.5289	0.8257	0.0013	−0.0606	−0.1414	−0.5703	0.1856	−0.0419	​	0.1984
Saikolignanoside A	X1	−0.0416	​	−0.0234	0.0485	0.0062	−0.0336	0.0203	0.0047	−0.4122	−0.3894
	X2	−0.4130	−0.0024	​	0.0159	−0.0003	−0.0113	0.0093	0.0673	0.0022	0.0807
	X3	0.1459	−0.0138	−0.0450	​	−0.0271	0.0020	−0.0392	0.3616	−0.0278	0.2106
	X4	−0.0367	0.0071	−0.0039	0.1076	​	0.0200	−0.0809	0.3751	0.1506	0.5755
	X5	−0.0428	−0.0327	−0.1092	−0.0067	0.0171	​	0.1003	−0.1201	−0.3589	−0.5102
	X6	0.2502	−0.0034	−0.0154	−0.0229	0.0119	−0.0172	​	−0.2024	−0.1234	−0.3727
	X7	0.5199	−0.0004	−0.0535	0.1015	−0.0265	0.0099	−0.0974	​	0.0373	−0.0290
	X8	−0.4977	−0.0345	0.0018	0.0082	0.0111	−0.0309	0.0620	−0.0390	​	−0.0212
Isochlorogenic acid A	X1	0.5224	​	0.0017	0.1654	0.0645	0.0297	0.0353	−0.0035	−0.4927	−0.1997
	X2	0.0293	0.0296	​	0.0542	−0.0035	0.0100	0.0162	−0.0494	0.0026	0.0596
	X3	0.4976	0.1736	0.0032	​	−0.2797	−0.0017	−0.0681	−0.2658	−0.0333	−0.4718
	X4	−0.3795	−0.0888	0.0003	0.3668	​	−0.0176	−0.1405	−0.2757	0.1800	0.0246
	X5	0.0378	0.4102	0.0077	−0.0229	0.1769	​	0.1741	0.0883	−0.4290	0.4053
	X6	0.4343	0.0425	0.0011	−0.0780	0.1227	0.0151	​	0.1487	−0.1475	0.1047
	X7	−0.3821	0.0048	0.0038	0.3461	−0.2738	−0.0087	−0.1691	​	0.0446	−0.0523
	X8	−0.5950	0.4327	−0.0001	0.0278	0.1148	0.0272	0.1077	0.0286	​	0.7387
Isochlorogenic acid C	X1	0.0432	​	−0.0088	−0.0281	0.0030	0.2957	0.0226	−0.0045	−0.1823	0.0977
	X2	−0.1551	0.0024	​	−0.0092	−0.0002	0.0996	0.0104	−0.0640	0.0010	0.0400
	X3	−0.0845	0.0143	−0.0169	​	−0.0129	−0.0174	−0.0436	−0.3440	−0.0123	−0.4327
	X4	−0.0175	−0.0073	−0.0014	−0.0623	​	−0.1756	−0.0900	−0.3568	0.0666	−0.6269
	X5	0.3766	0.0339	−0.0410	0.0039	0.0081	​	0.1115	0.1143	−0.1587	0.0720
	X6	0.2782	0.0035	−0.0058	0.0133	0.0056	0.1510	​	0.1925	−0.0546	0.3055
	X7	−0.4945	0.0004	−0.0201	−0.0588	−0.0126	−0.0870	−0.1083	​	0.0165	−0.2698
	X8	−0.2201	0.0357	0.0007	−0.0047	0.0053	0.2716	0.0689	0.0371	​	0.4146

In addition, precipitation in April exerted a substantial positive effect on the contents of eleutheroside B and saikolignanoside A, but a strong negative effect on the content of isochlorogenic acid C. The direct path coefficients were −0.3372, −0.0367, and −0.0175, respectively, while the indirect path coefficients were 0.8107, 0.5755, and −0.6269, respectively. Precipitation in September exerted a strong positive effect on the contents of isochlorogenic acid A and isochlorogenic acid C, and a strong negative effect on the contents of chlorogenic acid and saikolignanoside A. The direct path coefficients were 0.0378, 0.3766, −0.7909, and −0.0428, respectively, and the indirect path coefficients were 0.4053, 0.0720, 0.3243, and −0.5102, respectively. Precipitation in October exerted a strong positive effect on the contents of isochlorogenic acid A and isochlorogenic acid C, with direct path coefficients of 0.4343 and 0.2782, and indirect path coefficients of 0.1047 and 0.3055, respectively. Precipitation in December exhibited a positive influence on the contents of eleutheroside B and saikolignanoside A, while demonstrating a negative effect on the contents of isochlorogenic acid A and isochlorogenic acid C. Precipitation in February showed a positive impact on the content of eleutheroside B and a negative influence on isochlorogenic acid C. Solar radiation in April exerted a negative effect on the content of saikolignanoside A.

### 3.9 Integrated analysis

The above results indicate that the accumulation of eleutheroside B, chlorogenic acid, and saikolignanoside A was correlated with precipitation in February, April, September, October, and December, solar radiation in April, as well as altitude and latitude within the studied range. The content of isochlorogenic acid A was correlated with precipitation in September and December. Similarly, the accumulation of isochlorogenic acid C was correlated with precipitation in September, April, and December.

## 4 Discussion

As a plant with both medicinal value and commercial development potential, *D*. *dentiger* has seen increasing demand in recent years in the fields of traditional Chinese medicine preparations, health products, and clinical applications. *D. dentiger* contains a variety of bioactive constituents, including flavonoids, alkaloids, phenylpropanoids, volatile oils, and terpenoids (Yang et al., 2020b). Modern pharmacological studies have shown that extracts from the roots of *D. dentiger* can effectively alleviate histopathological changes in the knee joints of model rats and regulate inflammatory responses (Yang et al., 2021a). Its phenylpropanoid compounds may exert anti-rheumatoid arthritis effects through inhibition of the NF-κB, Akt, and JNK signaling pathways (Yang et al., 2021b). In addition, phenolic derivatives in *D. dentiger* have been reported to exhibit significant COX-2 inhibitory activity and antioxidant properties (Yang et al., 2021a). Previous studies on this medicinal plant have primarily focused on chemical constituent identification and pharmacological activities such as anti-inflammatory effects. In contrast, the present study employs multivariate analysis to reveal a geographical trend in the content distribution of five major active constituents in the roots of *D*. *dentiger*: eleutheroside B, chlorogenic acid, saikolignanoside A, isochlorogenic acid A, and isochlorogenic acid C. We also established that their accumulation correlates with precipitation, solar radiation, and geographical coordinates in specific months. Building on the identification of these chemical constituents, this research further analyzes the statistical correlations between their content variation and geographical-climatic factors. The findings may provide a reference for subsequent, more in-depth studies on metabolic regulation, germplasm resource evaluation, or exploration of cultivation practices.

The compounds focused on in this study—eleutheroside B and saikolignanoside A (lignans), along with chlorogenic acid and isochlorogenic acids A/C (phenolic acids)—are all phenylpropanoid compounds in terms of chemical structure and share the phenylpropanoid metabolic pathway as a common upstream biosynthetic source (Dong and Lin, 2021; Li et al., 2017). This suggests that the regional differences in their content may be related to the differential regulation of this common metabolic pathway by environmental factors. The phenylpropanoid pathway is a core secondary metabolic pathway in plants. Starting from phenylalanine, it produces numerous substances with defensive and adaptive functions, serving as a “central metabolic hub” for plants to respond to environmental changes. The metabolic activity of this pathway is strongly regulated by environmental stress, and the activities of its key rate-limiting enzymes, such as phenylalanine ammonia-lyase (PAL), are highly sensitive to environmental signals (Dong and Lin, 2021).

Precipitation regulates the phenylpropanoid metabolic pathway through complex physiological and biochemical processes. First, water stress induces a burst of reactive oxygen species (ROS), which act as signaling molecules to directly or indirectly activate key pathway enzymes such as PAL and 4-coumarate-CoA ligase (4CL), thereby promoting the synthesis of phenolic acid precursors. This mechanism has been confirmed in various plants. For instance, under water stress, the activities and gene expression of PAL and 4CL are significantly induced in *Poncirus trifoliata* (Liu et al., 2025) and *Cynodon dactylon* (Zhao et al., 2025). Second, environmental signals dynamically and precisely regulate downstream metabolic flux through transcription factor networks. An increase in upstream flux does not necessarily lead to the proportional accumulation of all downstream products. Research indicates that transcription factor families such as MYB and bHLH can specifically regulate the expression of genes in downstream branches of the phenylpropanoid pathway, thereby directing carbon flow toward different end products. For example, Wang et al. found that the *FtMYB3* transcription factor induced by drought in Tartary buckwheat (*Fagopyrum tataricum*) can negatively regulate anthocyanin synthesis by competitively binding to bHLH proteins (Wang et al., 2022). In *Salvia miltiorrhiza*, *SmMYB36* has been shown to simultaneously promote tanshinone accumulation and inhibit phenolic acid synthesis (Liu et al., 2015). These findings provide a potential molecular explanation for the observations in this study—namely, that differences in precipitation patterns across different production areas lead to a metabolic flux biased either toward isochlorogenic acid (phenolic acids) or toward eleutheroside B (lignans). Specifically, the precipitation pattern specific to a production area may differentially regulate the expression profiles of transcription factors such as MYB in *D*. *dentiger*, thereby determining the preference for phenylpropanoid metabolic branches.

Interestingly, different medicinal plants exhibit varied responses to precipitation, providing an important reference for understanding the unique response pattern of *D*. *dentiger*. Existing studies confirm that water conditions can generally regulate the metabolic patterns of phenolic compounds in plants. For example, in grassland ecosystems, increased precipitation treatment significantly alters the content and composition of phenolic compounds in plants (Top et al., 2017). However, the accumulation patterns of specific chlorogenic acid compounds exhibit species specificity and highly precise regulatory logic. This specificity stems from the differential interpretation and integration of environmental signals by plants. For instance, an increase in altitude implies not only potential changes in precipitation but also represents a composite environmental gradient involving coordinated changes in temperature, light, soil nutrients (Pan et al., 2023). This composite gradient can trigger a reconfiguration of the plant’s internal metabolic networks. Consequently, closely related metabolites within the same plant, such as chlorogenic acid and its various isomers, may exhibit opposing responses to the same environmental signal. The observed phenomenon in *D*. *dentiger* demonstrates a specific decoding of composite environmental signals and precise regulation of phenylpropanoid flux: eleutheroside B correlates positively with precipitation in February and April, whereas isochlorogenic acid C shows a negative correlation during the same period, reflecting its distinct genetic program and survival strategy.

Solar radiation can influence the synthesis and accumulation of flavonoids, phenolics, and other bioactive components by activating various molecular signaling pathways to regulate gene expression. Different wavelengths of light, such as UV-A and UV-B, serve as discrete cues that trigger plant photoadaptation and photostress responses, thereby orchestrating metabolic regulation. Zha et al. found that UV-A radiation can promote the synthesis of most phenylpropanoids and terpenoids derived from the shikimate pathway and the methylerythritol phosphate (MEP) pathway (Zha et al., 2024). UV-B can activate its specific photoreceptor UVR8-mediated signaling pathway as well as non-UV-B-specific pathways (e.g., DNA damage signaling, defense, and wound signaling pathways) to regulate gene expression. The latter involves various signaling molecules such as MAP kinases, reactive oxygen species, salicylic acid, and nitric oxide (Jenkins, 2017). Altitude and longitude, as composite environmental gradients, are often accompanied by synergistic changes in multiple factors such as temperature, light, ultraviolet intensity, and atmospheric pressure. To cope with the combined stresses of low temperature and strong UV radiation resulting from these changes, plants typically shift resource allocation from primary metabolism to secondary metabolism. Zhang et al. found that with increasing altitude, the phenylpropanoid metabolic pathway in plants is enhanced, leading to a significant increase in the content of antioxidants such as flavonoids and phenolic acids (Zhang et al., 2025). Similarly, the Tibetan medicinal plant *Phlomoides rotata* can adapt to the high-cold and strong UV environment of high-altitude areas by dynamically adjusting the accumulation of secondary metabolites such as flavonoids and monoterpenoids (Wang L. et al., 2025).

Analysis of the chemical constituents in *D*. *dentiger* from Zhejiang and Jiangxi provinces revealed distinct accumulation patterns between the two regions. Samples from Jiangxi Province exhibited higher total contents of eleutheroside B, chlorogenic acid, and saikolignanoside A, whereas samples from Zhejiang Province showed higher total contents of isochlorogenic acid A and isochlorogenic acid C. This divergence is likely associated with differences in the geographic, climatic, and soil environments of the two locations. Although both provinces are situated within the subtropical monsoon climate zone, their specific climatic conditions vary. Zhejiang, characterized predominantly by plains and hills and located along the coast, experiences more pronounced influence from summer monsoons, resulting in higher precipitation in September compared to Jiangxi. In contrast, Jiangxi is surrounded by mountains and features an interlaced topography of hills and plains inland, exhibiting a distinct inland basin character where cold air tends to accumulate. This leads to higher precipitation in February and April compared to Zhejiang. By December, both provinces enter a period of low precipitation, with minimal differences between them. The observed variation in component accumulation may therefore be linked to this spatiotemporal distribution pattern of precipitation. Within the framework of environmental regulation of plant metabolism, soil acts as a critical interface linking precipitation and plant physiology. Beyond direct water availability, the characteristic soil differences between the two provinces may serve as important synergistic factors. Specific soil conditions can profoundly alter the composition of plant root exudates (Tong et al., 2025). Zhejiang widely distributes acidic red soils, which are high in iron and aluminum, conditions that can lead to the fixation of nutrients such as phosphorus and shape specific rhizosphere microbial communities. This soil characteristic, synergistically with the local precipitation pattern, may collectively promote the accumulation of phenolic acids such as isochlorogenic acid in *D. dentiger*. Conversely, the soil conditions in Jiangxi likely foster different nutrient availability and microbial environments, which may be more conducive to inducing the biosynthesis of lignan components such as eleutheroside B. The underlying mechanism likely involves interactive feedback within the “plant-soil-microorganism” system. Plant roots can shape their distinctive rhizosphere microbial communities by secreting primary and secondary metabolites, including phenolic acids (Vives-Peris et al., 2020). In turn, these colonizing microorganisms can feedback-regulate host physiology and metabolism through multiple pathways: beneficial rhizobacteria may produce phytohormones (e.g., auxins, cytokinins) or elicitors, systematically altering the plant’s defense status and the expression of genes related to secondary metabolism (Pieterse et al., 2014). Microbial activity directly influences the mineralization and availability of key nutrients such as nitrogen and phosphorus in the soil, thereby affecting the plant’s nutritional status and carbon-nitrogen metabolic balance. This fundamentally determines the total resources the plant allocates to secondary metabolism, including the phenylpropanoid pathway (Jacoby et al., 2017). Rhizosphere microorganisms can metabolize phenolic compounds secreted by plants, such as chlorogenic acid. This consumption directly alters the concentration of these compounds in the rhizosphere environment and may, via feedback mechanisms, influence the plant’s own synthesis rate (Hu et al., 2018). Therefore, the climate and soil conditions in different production areas such as Zhejiang and Jiangxi synergistically regulate the metabolism of *D. dentiger*.

The findings of this study can provide references for the resource conservation and standardized cultivation of *D*. *dentiger*. Some production areas in Zhejiang Province are potential high-quality regions for obtaining materials rich in isochlorogenic acids. Resource conservation and base site selection could prioritize native habitats or similar ecological areas with specific precipitation patterns and soil characteristics. Key periods for water regulation are identified as April (early growth stage), September and October (material accumulation stage), and December (dormancy stage). In artificial cultivation, precise water management during these periods could be considered as a mild “stress elicitor” to directionally enhance target active components. It is recommended to set the harvest period in the late autumn to early winter, after the main stress-induction and material accumulation processes, to obtain medicinal materials enriched with secondary metabolites.

However, several limitations of this study must be noted. First, the sample collection was primarily confined to several provinces in southern China, which may not fully represent the overall chemical diversity of this species across its entire natural distribution. Therefore, the observed patterns in compound content should be interpreted with caution, and future studies should expand the sampling scope to include populations from a broader geographical range. Secondly, although this study found a significant correlation between compound accumulation and environmental factors such as precipitation and solar radiation, its potential regulatory mechanisms, such as key enzyme activity and gene expression, still need further research.

In summary, this study revealed that the contents of eleutheroside B, chlorogenic acid, saikolignanoside A, isochlorogenic acid A, and isochlorogenic acid C in *D. dentiger* varied significantly among production areas. Precipitation in April, September, and December, as well as longitude, exert considerable influence on the accumulation of these compounds. In recent years, however, excessive harvesting of *D. dentiger* from the wild has led to a sharp decline in its natural resources. As a rare and endangered ethnomedicine of the She nationality, urgent attention is required for the conservation of its germplasm resources. The findings of this study provide valuable references for refined cultivation of *D. dentiger* in different production areas and contribute to its sustainable development and utilization.

## Data Availability

The original contributions presented in the study are included in the article/supplementary material, further inquiries can be directed to the corresponding authors.
